# Quality Characteristics of Fresh Date Palm Fruits of “Medjoul” and “Confitera” cv. from the Southeast of Spain (Elche Palm Grove)

**DOI:** 10.3390/foods12142659

**Published:** 2023-07-10

**Authors:** Clara Muñoz-Bas, Nuria Muñoz-Tebar, Laura Candela-Salvador, José A. Pérez-Alvarez, José M. Lorenzo, Manuel Viuda-Martos, Juana Fernández-López

**Affiliations:** 1 IPOA Research Group, Centro de Investigación e Innovación Agroalimentaria y Agroambiental, Universidad Miguel Hernández (CIAGRO-UMH), Ctra. Beniel Km 3.2, 033121 Orihuela, Spain; clara.munozb@umh.es (C.M.-B.); nmunoz@umh.es (N.M.-T.); laura.candela03@goumh.es (L.C.-S.); ja.perez@umh.es (J.A.P.-A.); mviuda@umh.es (M.V.-M.); 2 Centro Tecnológico de la Carne de Galicia, Avd. Galicia 4, 32900 San Cibrao das Viñas, Spain; jmlorenzo@ceteca.net

**Keywords:** date fruit, Confitera, Medjoul, food quality, polyphenol composition, antioxidant properties

## Abstract

The quality characteristics (physical, techno-functional, and chemical) of date fruits (at the tamar stage) of two cultivars (“Confitera”, autochthonous and unknown vs. “Medjoul”, widely distributed and well-known), grown in the Southeast of Spain (Elche palm grove) were evaluated in order to reinforce decisions aimed at organizing the production of fresh dates from Elche by selecting the most profitable cultivar. Morphologically, Confitera dates were longer and with higher pulp yield than Medjoul dates (4.58 cm vs. 3.88 cm, and 84% vs. 78%, respectively) (*p* < 0.05). Nutritionally, both dates are a good source of carbohydrates (total sugars (43–48%) and dietary fiber (20–22%)), with small amounts of fat and proteins. The main free sugars in dates from both cultivars were glucose and fructose (reducing sugars). The most abundant mineral found in both date fruits were K, followed by Ca or Mg (depending on the cultivar; Ca in Medjoul and Mg in Confitera). Confitera dates showed a higher total antioxidant activity than Medjoul, corresponding with their higher (*p* < 0.05) content in polyphenolic compounds, mainly flavonoids (catechin and epicatechin predominantly). Confitera dates should be promoted in this region not only for their contribution to sustainable agricultural development and biodiversity, but also for their higher overall quality.

## 1. Introduction

The date, a fruit of the date palm tree (*Phoenix dactylifera* L.) that originated in the ancient world (dating back to before 4000 B.C.), is of great cultural, social, environmental, livelihood, and economic importance in many countries, being particularly important in arid and semiarid regions with marginal agriculture, where it is one of the pillars on which they are based. In these regions, it has been considered a relevant subsistence crop without misleading its importance as a cultural legacy in Arabic countries and some Islamic ones. From its origin (ancient Mesopotamia or western India), date culture has been spread to other areas of the world such as South Asia, North Africa, around Mediterranean, Australia, Mexico, and United States. All these areas have in common a hot and dry climate (long and hot summer, little rain and low humidity), and have typical characteristics of desert and semi-desert areas that can be found in larger zones due to current climate change [1,2,3]. It is the case of the Southeast of Spain (Elche, Alicante) where the largest palm grove in Europe and one of the northern and largest in the world (approx. 500 hectares with more than 200,000 palm trees’ specimens, whose production in 2020 was 1375 tons) is located [4], declared in 2000 a World Heritage Site by UNESCO.

Traditionally, dates have been a product of exchange and domestic trade, which means that they have been mainly consumed in productive countries, but this has undergone a change over time, and currently there is a very active trade of dates from the production areas to Europe and America. This date export market is essentially supplied by dry and semi-soft dates, which are easier to preserve than fresh dates that are rich in water [5,6]. However, fresh dates are healthier and are most appreciated by current consumers. In addition, although it is believed that more than 2000 cultivars of dates exist [7], only a few have importance due to their agronomic characteristics and fruit quality, being Medjoul cv. one of the most valuable in the European trade. The Medjoul cv. has distinct advantages over other cultivars, such as high yields and quality fruits, huge value in the international market, and great nutritional value [8]. This is the cultivar that has been mainly cultivated in Elche palm grove although during last years a not well-known autochthonous Confitera cv. is being promoted due to it is completely adapted to this particular habitat, ecosystem, and region and also a way to preserve biodiversity. At this time, Medjoul and Confitera cv. are the only cultivars with commercial potential in this area.

Nutritionally, date fruits are an important (and cheap) source of carbohydrates (total sugars: 50–80 g/100 g dw, and dietary fiber: 6.4–11.5 g/100 g dw (mainly insoluble dietary fiber)) with a low content in fats (0.2–0.5 g/100 g dw) and proteins (1.6–4.7 g/100 g dw) [1]. In addition, their content in micronutrients (minerals such as K, Ca, Mg, Na, Mn, Zn and Fe) and vitamins (B-complex and vit. C) and other bioactive compounds (phenolic compounds, anthocyanins, sterols, and carotenoid) [9] give them a high-added value mainly related to their functional and healthy properties [1,2,3,10,11]. However, this composition highly depends on the ripening stage, cultivar, growth region, and climatic conditions [12,13]. Through their ripeness stages (hababuk, kimri, khalal, rutab and tamar), date fruits undergo relevant variations in the color, texture, taste, and chemical composition [14]. At the final stage of ripening (tamar), the fruit is completely ripened, and the moisture is also reduced, which is the best moment for its consumption.

From a commercial point of view, the Medjoul cultivar is the dominant one in the palm grove of Elche, leaving the autochthonous cultivars at a disadvantage both commercially and scientifically. The present study will provide scientific data that will give added value to the autochthonous Confitera cv., which is totally adapted to the edaphoclimatic conditions of this special growing area. The objective of this work was to compare fresh dates from Medjoul and Confitera cv. of the Elche palm grove at the tamar stage, by means of their morphological characteristics, physicochemical properties, nutritional composition, bioactive compounds content and antioxidant properties. These data will contribute to a better understanding of their overall quality and, to support decisions aimed at organizing the production of fresh dates from Elche by selecting the most profitable cultivars.

## 2. Materials and Methods

### 2.1. Raw Materials

Date fruits of cv. Medjoul and Confitera were harvested (manually picked, from October 2021–February 2022) at the tamar stage from date palm trees (7000 from each cultivar) located in the Elche palm grove (Elche, Alicante, Spain) by specialized staff and transported under refrigerated conditions to the IPOA laboratory of the Orihuela Campus at the Miguel Hernández University (Orihuela, Alicante, Spain). The average sample used in this work was 45 kg from each cultivar.

### 2.2. Morphological Characteristics

Morphological parameters consisting of date fruit weight, length, and width were measured from 20 healthy and uniform date fruits from each cultivar (Medjoul and Confitera) that were randomly selected. After that, each of the selected dates was manually peeled and the seed was separated; then, the weights of skin, pulp and seed were recorded. The fresh weights of the date, seed, and pulp were determined using an analytical balance PLI-360-3M (Kern & Sohn, Balingen, Germany), and the length and width determined using a Vernier caliper. Mean values and standard deviations were recorded.

### 2.3. Physicochemical Properties

The pH of date fruit was determined using a calibrated pH-meter GLP21 (Crison, Barcelona, Spain) on a water suspension (0.5 g sample with 50 mL deionized water blended for 2 min).

The external color of date fruit was measured using a CM-700 Minolta spectrophotocolorimeter (Minolta, Osaka, Japan) in CIELAB mode under CIE Standard Illuminant D65 and observation angle 10°. CIELAB coordinates (lightness (L*), red/green (a*) and yellow/blue (b*)) were obtained from which the psychophysical magnitudes Chroma (C* = (a*^2^ + b*^2^)^1/2^) and Hue (H* = arctg b*/a*) were calculated. Color differences (ΔE* = (ΔL*^2^ + Δa*^2^ + Δb*^2^)^1/2^ between Medjoul and Confitera dates were also calculated.

### 2.4. Technofunctional Properties

The water-holding capacity (WHC), oil-holding capacity (OHC) and swelling capacity (SWC) of date fruit samples were measured following methods described by López-Marcos et al. [15]. To determine the WHC of the date fruit samples, a centrifuge tube containing 500 mg of date fruit was supplemented with 10 mL of ultrapure water. The tubes were then stored at 25 °C for 18 h. Subsequently, centrifugation was carried out using a centrifuge (Nahita 2652, Alicante, Spain) at 3000 rpm for 20 min at the same temperature. Following centrifugation, the supernatant was discarded, and the remaining pellet was weighed. The WHC value for each sample was calculated as the weight of water retained by 1 g of the corresponding date fruit sample. For OHC, a similar procedure previously described for WHC was applied but using 5 g of sunflower oil with 500 mg of date fruit samples, with the results expressed as the weight of oil held by 1 g of corresponding date fruit samples. For the SWC, date fruit samples (200 mg) were weighed in a 10 mL tube and the volume occupied by the date fruit sample was recorded before adding 5 mL ultrapure water. The mixture was stirred to eliminate any trapped air bubbles and then left on a level surface at room temperature for 24 h, allowing the sample to settle. The volume (mL) occupied by the hydrated date fruit samples was measured, and SC was expressed as milliliters per gram of date fruit samples.

### 2.5. Proximate Composition

Total ash (AOAC 923.03), protein (AOAC 981.10), fat (AOAC 991.36), dietary fiber (AOAC 985.29) and moisture content (AOAC 925.45) of date fruits were determined (in triplicate) using AOAC methods [16]. Total sugars content was estimated by difference, subtracting the sum of the other components (moisture, fat, protein, ash and TDF) from the total (100%).

### 2.6. Organic Acids and Sugars Profile

#### 2.6.1. Extraction of Organic Acid and Sugars

Fifty mL of ultrapure water, acidified with *ortho*-phosphoric acid (0.1% *v*/*v*), were added to 1 g of the Confitera and Medjoul date fruit paste and stirred at room temperature for 24 h. Then, solutions were homogenized at 20,000 rpm for 2 min in a homogenizer (Ultra-Turrax T25 BASIC, IKA-Werke GmbH & Co. KG, Staufen, Germany) and heated at 80 °C for 1 h under constant stirring. After that, the samples were centrifuged at 6500× *g* for 10 min at 4 °C and the supernatants were filtered through a 0.45 μm filter.

#### 2.6.2. HPLC Analysis

Organic acids and sugars were quantified according to Melgarejo-Sánchez et al. [17]. The HPLC system used was a Hewlett-Packard 1100 series model (Woldbronn, Germany). The samples (20 μL) were injected in a Supelco column (Supelcogel TM C-610H column 300 mm × 78 mm) and absorbance was measured at 210 nm using a diode-array detector (DAD G-1315A). The elution buffer was *ortho*-phosphoric acid in water (0.1% *v*/*v*) with an isocratic flow rate of 0.5 mL/min. These same HPLC conditions (elution buffer, flow rate and column) were employed for the determination of sugars. Though, the detection was carried out by means of a refractive index detector (RID G1362A). Standards of organic acids, monosaccharides and oligosaccharides were obtained from Supelco (Sigma-Aldrich, St. Louis, MO, USA). Peaks were identified by comparison with theretention time of the standards, and quantified by regression formula obtained with the standards.

### 2.7. Mineral Composition

The determination and quantification of mineral content of the lyophilized date fruits samples (Freeze dryer Alpha 2–4, Martin Christ Gefriertrocknungsanlagen GmbH, Germany) were carried out by Inductively Coupled Plasma Mass Spectrometry (ICP-MS-2030) (Shimadzu, Kyoto, Japan). Mineral content was quantified after digestion with 67% nitric acid and 33% hydrogen peroxide by a microwave system. In order to estimate the content of the concentrations of macro- and micro-elements, calibration standards were prepared. The final value per sample was the average of 3 reads, and mineral content was expressed as mg/100 g dry weight of date fruit.

### 2.8. Polyphenol Composition

The extraction of polyphenolic compounds was conducted following the methodology described by Genskowsky et al. [18]. To minimize any potential interference from the sugar content in the samples during chromatographic analyses, a C-18 Sep-Pak cartridge was utilized. Prior to loading the extracts onto the cartridge, it was conditioned with 3 mL of methanol, 3 mL of ultrapure water, and 3 mL of hydrochloric acid (10 mM). Subsequently, the cartridge was washed with 5 mL of ultrapure water. The final step involved eluting the cartridge with 3 mL of acidified methanol (0.1 g/L formic acid). The resulting extracts were carefully preserved at −40 °C until HPLC analysis.2.8.2. HPLC analysis.

For the determination of polyphenolic compounds, a LC-MS/MS 8050 High Performance Liquid Chromatography triple quadrupole mass spectrometer (Shimadzu, Kyoto, Japan) was used. The ISE source was operated with a nebulizer gas flow of 3 L/min, drying gas flow of 10 L/min, a desolvation line (DL) temperature of 250 °C, and a heat block temperature of 400 °C. Selected ion monitoring (SIM) with a collision energy of −35 V and full MS scans in positive mode between 100–1000 m/z. The chromatographic separations were performed with a Mediterranean SEA18 column (10 mm L × 0.21 mm i.d., 2.2 μm particle size, Teknokroma, Barcelona, Spain) maintained with a temperature at 50 °C. The mobile phase A consisted of 0.1% formic acid in ultrapure water and the mobile phase B consisted of 0.1% formic acid in acetonitrile. The gradient elution was 0–2 min 5% B, 2–10 min 95% B, 10–11 min 95% B, 11–12 min 5% B, and 12–16 min 5% B with a flow rate of 0.400 mL/min and injection volume of 10 μL. External standards were used for the quantification of polyphenolic compounds; a stock of 200 mg/L was made and from it a calibration plot of concentrations 0.1, 0.3, 0.5, 0.8, and 1 mg/L was obtained. Labsolutions LCMS software Ver. 5.98 (Shimadzu) was used for instrument control and data-processing. All analyses were done in triplicate.

### 2.9. Antioxidant Properties

The antioxidant properties of date fruits were assessed usingfourdifferent antioxidant assays. DPPH assay was performed following the method proposed by Brand-Williams et al. [19] TEAC-ABTS assay was established following the method proposed by Gullón et al. [20]. Ferric reducing antioxidant power (FRAP) was assessed by means of potassium ferricyanide-ferric chloride method described by Oyaizu [21]. Ferrous ions chelating activity (FIC) was determined establishing the inhibition of Fe^2+^ -ferrozine complex formation after adding to test material Fe^2+^ by means of the method described by Mahdavi et al. [22]. Absorbance values were measured on a spectrophotometer at 517 nm (for DPPH assay), 734 nm (for TEAC-ABTS assay), 700 nm (for FRAP assay) and 562 nm (for FIC assay). Trolox was used as the reference standard for DPPH, TEAC-ABTS and FRAP assays, and EDTA for FIC assay. The results were expressed as μg Trolox Equivalents/g of date fruit in the case of DPPH assay, and as mg Trolox Equivalents/g of date fruit in the case of ABTS and FRAP assays. For the FIC assay, the results were expressed as μg Ethylenediaminetetraacetic Equivalents (EDTAE)/g of date fruit.

### 2.10. Statistical Analysis

All determinations were made in triplicate and results are shown as mean ± standard deviation. Comparison was conducted using the one-way Analysis of Variance (ANOVA) at a confidence level of 95%. These analyses were carried out with the statistical program SPSS for Windows v. 27.0 (SPSS Inc., Chicago, IL, USA).

## 3. Results and Discussions

### 3.1. Morphological Characteristics

**Figure 1:**
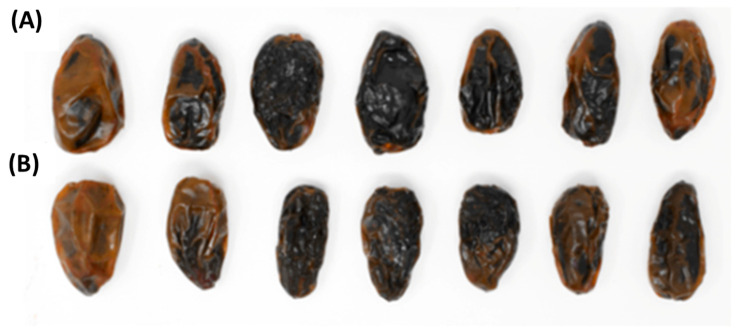
Date fruits at the tamar stage of Medjoul (**A**) and Confitera (**B**) cultivars.

Figure 1 shows some date fruits atthe tamar stage of both cultivars, Confitera and Medjoul, while their morphological characteristics are shown in Table 1.

These results indicate that the dates of the Confitera cv. are longer (*p* < 0.05) than those of Medjoul. Although there are no differences in the total weight of the dates of both cultivars, the Confitera date fruits have a higher proportion (*p* < 0.05) of pulp than seed in comparison with Medjoul date fruits, which results in a higher pulp yield (84% for Confitera cv. vs. 78% for Medjoul cv.), since the edible part is the pulp being the seed considered a coproduct. The morphological characteristics of Medjoul dates are within the range of the data reported for this cultivar in different growing areas [8,23,24]. No bibliographic references have been found on the morphological characteristics of Confitera dates at the tamar stage. It is also important to note that these properties are highly dependent on the ripening stage, and it is not during the tamar stage (fully ripe stage) that the highest values (weigh, size and yield) are reached [24,25,26]. These authors reported that date fruits grew considerably until the khalal stage, mainly due to faster cell division and elongation processes, and then, from the halal to tamar stage a decreasing trend was observed, due to fruit shrinkage after reaching the final tamar stage. According to the quality standards of date fruits applied in the international scale reported by Meligi and Sourial [27], in reference to the length, width, total weight, pulp weight and seed weight/date weight (%), all date fruits would receive the highest evaluation (good character), except Medjoul dates for the length that would be evaluated as medium quality (3.5–4 cm; acceptable).

### 3.2. Physicochemical Properties

The pH is one of the most important parameters affecting their processing and storage quality. The results shown in Table 2 reveal higher pH values (*p* < 0.05) in Confitera than in Medjoul dates. Both pH values are within the range reported for fruit dates at the tamar stage (5.2–6.3) [28,29]. In both cases, pH values were higher than 5, and the limit value associated with an acidic taste, evaluated as bad character by the consumer. The acidic nature of date fruits is due to the natural organic acids originating from date fruit including succinic, malic, tartaric and ascorbic acid (see Section 3.5).

Color plays a key role in the quality index and marketing value of fruits. In the case of date fruits, intense color variations are closely related with cultivar and ripening progresses [14,30,31]. Nevertheless, different date cultivars exhibit their own color upon ripening [31]. During ripening, evident changes in the color of date fruits results from the degradation of chlorophyll, marking the transition from one stage to another. Date fruits are rich in carotenes, orange-yellow to red crystalline pigments (fat-soluble) which are responsible for their bright and typical color. Furthermore, it has been reported that during ripening the concentration of chlorophyll decreases but the carotenoid concentration does not improve or even decrease, which is represented by the presence of yellow-brown color in dates [14,32]. At the last ripening stage (tamar), date fruits are usually less luminous and darker than at the first stages, which is related to the loss of water [33]. Color parameters of the surface of Medjoul and Confitera date fruits at the tamar stage are shown in Table 2. Regarding color coordinates, Confitera dates showed higher a* and b* values (*p* < 0.05) and a similar lightness (*p* > 0.05) than Medjoul dates. In any case, as the color differences (ΔE*) were lower than three units (1.16 ± 0.02), they cannot been detected by the human eye. Confitera dates showed a more saturated color (*p* < 0.05) than Medjoul dates although the hue of the dates from both cultivars was the same (*p* > 0.05), corresponding to a red-orange hue [34]. In addition to the content and/or proportions of natural pigments (chlorophylls, carotenes, anthocyanins, etc.), the color of dates is also influenced by the development of non-enzymatic browning reactions (Maillard reaction and caramelization) promoted by their high content of reducing sugars (see Section 3.5).

### 3.3. Techno-Fucntional Properties

**Figure 2:**
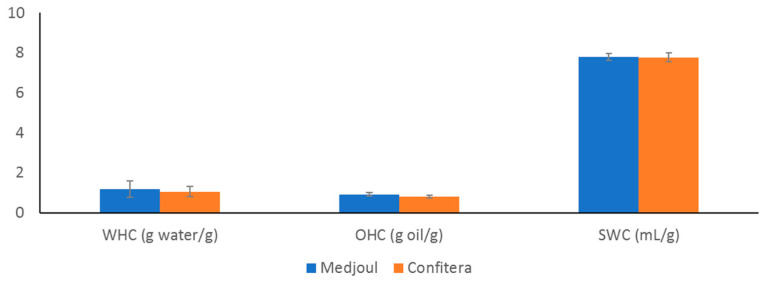
Techno-functional properties of Spanish-grown Medjoul and Confitera date fruits.

Techno-functional properties (WHC, SWC and OHC) of Confitera and Medjoul date fruits are shown in Figure 2.

The hydration properties (WHC and SWC) of food materials are important not only for its physiological role but also for their influence on the techno-functional properties of the food. WHC and SWC provide information regarding the hydration capacity of the food matrix and give insights into its behavior during gut transit and food processing [15,35]. It has been reported that food matrices with good hydration properties (high WHC and SWC) could be added as a functional ingredient in the development of functional foods due to their physiological effect during digestion, absorbing water in the gut contributing to stool bulking [36,37]. However, other authors have reported that a high affinity to water could show some negative effect on the texture and shelf life of the final food [36,38]. Medjoul and Confitera dates showed similar (*p* > 0.05) hydration properties (WHC and SWC), being SWC values higher (*p* < 0.05) than corresponding WHC values. It must be noted that the WHC is the ability of a material to retain water when subjected to an external force (centrifugation or pressure), so it consists of the sum of bound water, hydrodynamic water and, mainly, physically trapped water. However, the SWC represents the amount of water that can be absorbed, and it is regarded as an indicator of a structure’s aptitude to spontaneously absorb water when in contact with a constantly moist surface or when immersed in water. For this reason, it is expected that SWC showed higher values than WHC. In any case, it could be said that Medjoul and Confitera date fruits at the tamar stage have no good hydration properties, in comparison with other vegetable matrices [39,40]. No data have been found on the hydration properties of date pulp as such, but there are much data referring to extracts rich in dietary fiber obtained from the date pulp of several cultivars [29,41,42] and even from the bagasse obtained after the extraction of date juice [43]. In both cases, the values reported are higher than those obtained here, but this is due to the high hydration properties attributed to dietary fiber, which in these extracts is in larger proportions than those present in fresh date pulp (raw material).

The OHC is also an important technological property that reflects the amount of oil retained in the matrix after incubation with oil and centrifugation and depends on the chemical and physical structure of polysaccharides, their surface properties and porosity [15,44]. Again, there were no differences between (*p* > 0.05) Medjoul and Confitera dates for OHC. Similar OHC values have been reported for some vegetable matrices such as passion fruit, pineapple, guava, or apples [40].

### 3.4. Proximate Composition

The results of the proximate composition of Confitera and Medjoul dates are shown in Table 3. Total sugars were the predominant fraction in date pulp from both date cultivars, followed by moisture and total dietary fiber, with small amounts of proteins, fats and ashes.

Medjoul dates showed higher (*p* < 0.05) moisture content than Confitera. It is widely documented that the moisture content of date pulp decreases throughout the different stages of ripening, with the last stage (tamar) having the lowest moisture content [1]. Depending on cultivar and growing conditions (place, climate, etc.) large differences in the moisture content of date pulp have been reported, ranging from 9 to 32% [28,45], being our data within this range and close to the highest moisture content. Protein, fat, and ash occur in small amount in date fruits, observing that Confitera dates had lower (*p* < 0.05) fat and higher (*p* < 0.05) protein content than Medjoul, but the same ash content (*p* > 0.05). Although the protein content in dates is not too relevant, they have been reported to contain high amount of some essential amino acids [3,12], some of which are not present in the most popular fruits, such as oranges, apples and bananas. It has been reported that the fat, protein and ash content decreases throughout the ripening process of the dates, reaching the lowest values at the end (tamar stage) [46]. On the contrary, simple carbohydrates content increases through ripening [28]. Medjoul dates showed lower (*p* < 0.05) total sugar content than Confitera, being this content, in both cases (63.8 and 64.9 mg/100 g dw., respectively), is lower than the one reported for other cultivars at tamar stage (73–88 g/100 g dw) [29,45]. There were no significant differences (*p* > 0.05) in TDF content between Medjoul and Confitera dates, being these results are higher than those reported for dates of different cultivars and the degree of ripeness (6.4–15.7 g/100 g) [47,48]. As well as total sugar concentration, TDF content is also dependent on the date cultivar, ripening stage, and climatic and growing conditions [47,49,50]. On the contrary to that reported for total sugars, TDF content decreases with the fruit transition from kimri to tamar stages, which has been attributed to the gradual enzymatic breakdown of these substances to more soluble compounds that soften the fruit [3,45,50]. In any case, even at the tamar stage, date pulp can be considered a good source of TDF (with higher IDF content than SDF) [1,29].

### 3.5. Sugars and Organic Acids

**Figure 3:**
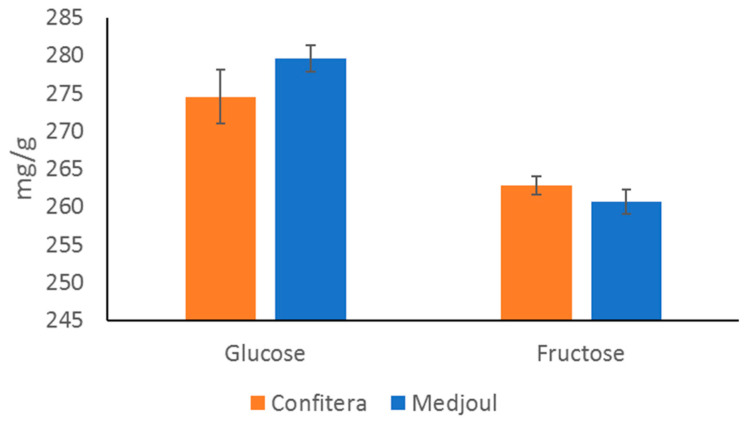
Sugar content (mg/g) of Spanish-grown Medjoul and Confitera date fruits.

**Figure 4:**
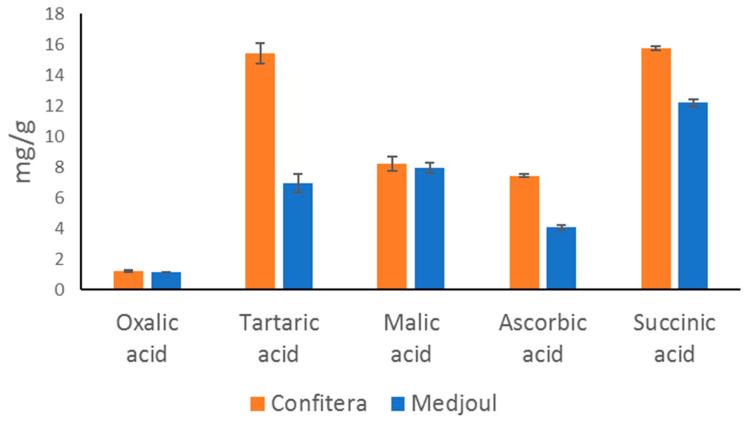
Organic acid content (mg/g) of Spanish-grown Medjoul and Confitera date fruits.

While sugars are responsible for the sweetness of date fruits, organic acids are responsible for their taste, contributing to sourness and modulating their sweetness. The content of the sugars and organic acids of Confitera and Medjoul date fruits is shown in Figure 3 and Figure 4, respectively.

Date pulp is characterized by its high sugar content, with the major sugars being glucose (the highest amount in Confitera dates, *p* < 0.05) and fructose (with no differences between cultivars, *p* > 0.05) in a similar proportion. Similar values of reducing sugars have been reported for Medjoul dates grown in Mexico [8]. These two reducing sugars have also been reported as the main sugars in several date cultivars (Alligh, Boufeggous, Goundi, Ikhouat, Kenta, Lagou, Touzerzaillet, Ajwa, and Tranja, among others) at the tamar stage [29]. It is important to compare sugar content among cultivars but at the same ripening stage because it has been reported a relevant increase of sugars during ripening [29,48,49], being that the sugars are at the highest concentration during the tamar stage. Alternatively, other authors have reported higher amounts of sucrose than reducing sugars in several varieties (Sokari, Mabroom and Deglet Noor), but also in this case, their proportion was depending on ripening stage. They reported that sucrose was present only at the khalal and rutab stages (and not at the tamar stage) [49], in agreement with our results. The reason given by these authors to explain the sudden drop in sucrose and the increase in reducing sugars at the tamar stage was the rising activity of the invertase enzyme implied in the hydrolysis of sucrose into fructose and glucose. Regarding this, it could be said that these two sugars are responsible for the sweetness of the fruit and also for their softness (along with moisture) and they would even contribute to fruit color through Maillard and caramelization reactions [31].

Four major organic acids were detected in Confitera and Medjoul date fruits, mainly succinic, tartaric, malic and ascorbic acid with a smaller concentration of oxalic acid. Confitera dates showed higher (*p* < 0.05) amounts of tartaric, succinic and ascorbic acid than Medjoul dates and similar content of oxalic and malic acids (*p* > 0.05). All these organic acids have been found in dates from several cultivars, ripening stages, and growth conditions although with differences in their proportion [31,39,48]. In general, it could be said that total acidity should decrease during ripening, but with significant changes in the predominance of specific organic acids [31]. In addition to their effect on date taste, organic acids are also important for date quality due to their potential effects as preservatives (antimicrobial agents).

### 3.6. Mineral Composition

Although it has been reported that mineral content in date fruits decreases during ripening [28,51], even at the last ripening stage (tamar), dates can be considered, nutritionally, a significant source of important minerals in the diet. The mineral content of Medjoul and Confitera dates is shown in Table 4. The most abundant mineral found in both date fruits were potassium, followed in descending order by calcium or magnesium (depending on the cultivar; calcium in Medjoul vs. magnesium in Confitera), sodium, iron, zinc, copper and manganese. The same order has been reported by other authors in date fruits of different cultivars and growing places [8,29,51,52] confirming that date fruits are rich in most of the macroelements but poor in microelements. Confitera dates showed higher amounts (*p* < 0.05) of iron, potassium, magnesium, manganese, sodium and zinc than Medjoul dates and similar amounts (*p* < 0.05) of calcium and copper. What is interesting is the low sodium:potassium ratio of both cultivars, which is in line with the current dietary recommendations to decrease the risk of cardiovascular diseases [53]. Comparing the mineral content of these Medjoul dates with data from the same cultivar but growing in other countries, several differences have been noted: Spanish dates showed lower content in potassium than Medjoul dates from Mexico or Morocco (851 and 849 mg/100 g dw, respectively) [8,52], but higher amount of magnesium, calcium, sodium, zinc and copper than Moroccan dates (68, 54, 11, 0.37 and 0.34 mg/100 g dw, respectively) [52].

### 3.7. Phenolic Composition and Antioxidant Activity

Date fruits can be considered a good source of antioxidant compounds, mainly polyphenolic compounds, especially in the earlier edible ripening stages. The nature, formulation, and distribution in dates vary with different factors such as variety, ripening stage, location, and environmental conditions [1,12,13,54]. The phenolic composition of Medjoul and Confitera date fruits growing in Spain is shown in Table 5. Eight polyphenolic compounds (seven flavonoids and one phenolic acid) were identified in Confitera dates and only six (five flavonoids and one phenolic acid) in Medjoul dates.

Both flavonoids and phenolic acids have been reported as the main polyphenol groups found in date fruits [55]. More than 13 different flavonoid glycosides and 19 isomeric forms of flavonoids have been identified in date fruits by several authors [12,13,49,56]. In addition, cinnamic acid derivatives are usually found in date fruits from several varieties [57,58]. Tassoult et al. [49] analyzed the phenolic profile of several date varieties from Algeria, reporting that although compound patterns exhibited significant variations based on the cultivars, solvent used, and the stage of ripening, caffeic acid was the major phenolic acid, and catechin and luteolin the major flavonoids found in all samples. Confitera dates presented higher amount of epicatechin, catechin, eriocitrin, hesperidin and rutin, but a lower amount of luteolin-7-O-glucoside and isoquercitrin, than Medjoul dates (*p* < 0.05). There were no significant differences (*p* > 0.05) in the content of caffeic acid between both date fruits. Caffeic acid has been reported as one of the major phenolic compounds found in dates of different varieties and locations [10,59,60]. As has been previously commented, each cultivar of date synthesizes characteristic flavonoids that, depending on different environmental conditions (temperature, hours of light), sanitary conditions or growing conditions, expressed in higher or lower concentrations, which could explain the differences found in polyphenol composition between Medjoul and Confitera dates.

The total antioxidant activity of Confitera and Medjoul dates assessed by means of four different methods (DPPH, ABTS, FRAP and FIC) is shown in Table 6.

These methods have been trying to cover the different mechanisms implied in the antioxidant activity (scavenging activity, reducing power and metal chelating). Confitera dates showed higher (*p* < 0.05) antioxidant values than Medjoul dates for the DPPH, FRAP and FIC methods and similar (*p* > 0.05) values for the ABTS method. Several authors have reported good antioxidant activity of date fruits of several cultivars (Tantebouchte, Biraya, Degla Baidha, Deglet-Nour, Ali Ourached, Ghars, and Medjoul, among others) obtained from different countries (Algeria, Kuwait Oman, Iran, Saudi Arabian, USA, Mexico, etc.) [2,49,60,61]. Allaith [62] reported similar values for the antioxidant activity, using the FRAP assay, of Bahrain dates at the tamar stage. Most of these authors confirmed that the antioxidant potential of date fruits was significantly correlated with the total phenolic and flavonoid compounds present in these fruits. In this case, a similar trend can be observed because Confitera dates showed higher total antioxidant activity than Medjoul dates, corresponding with their higher content in polyphenolic compounds, mainly flavonoids. In any case, it cannot be forgotten that other compounds found in date pulp (anthocyanins, phytosterols, carotenoids, and selenium) could also contribute to its antioxidant properties [1,2,63]. In agreement with data previously reported, Spain-grown Confitera and Medjoul dates contain good amounts of different polyphenolic compounds which have shown in vitro antioxidant potential, making them not only a valuable food, but also a promising ingredient in the development of functional foods.

## 4. Conclusions

Based on the results, it could be said that the growth and production of date fruits of the autochthonous Confitera cultivar should be promoted in this region (Southeast of Spain: Elche palm grove) not only for their contribution to sustainable agricultural development and biodiversity, but also for their higher overall quality in comparison with date fruits from Medjoul cultivar growth in similar conditions. Confitera dates are longer and have higher pulp yield than Medjoul. Nutritionally, both dates are a good source of carbohydrates and dietary fibers, with a small amount of fats and proteins. The main free sugars in dates from both cultivars were glucose and fructose (reducing sugars). The most abundant minerals found in both date fruits were K, followed by Ca or Mg (depending on the cultivar; Ca in Medjoul and Mg in Confitera). In addition, although both cultivars are rich in polyphenols, mainly flavonoids, with remarkable in vitro antioxidant potential, Confitera dates show a higher number of polyphenol compounds (and at higher concentrations) than Medjoul dates. In general, it can be said that fresh Confitera and Medjoul dates from the Southest of Spain can be considered valuable foods and promising ingredients in the development of functional foods.

## Figures and Tables

**Table 1:** Morphological characteristics of Spanish-grown Medjoul and Confitera date fruits.

Date cv.	Length (cm)	Width (cm)	Total Weight (g)	Pulp Weight (g)	Skin Weight (g)	Seed Weight (g)
Medjoul	3.88 ± 0.36	2.14 ± 0.25	11.64 ± 3.85	9.08 ± 3.16	1.15 ± 0.37	1.23 ± 0.45
Confitera	4.58 ± 0.22	2.14 ± 0.17	13.12 ± 1.61	10.97 ± 1.48	1.09 ± 0.20	0.97 ± 0.17
*p* value	0.000	0.943	0.122	0.021	0.485	0.018

**Table 2:** Physicochemical properties [lightness (L*), red/green coordinate (a*), yellow/blue coordinate (b*), chroma (C*) and hue (H*)] of Spanish-grown Medjoul and Confitera date fruits.

Date cv.	pH	L*	a*	b*	C*	H*
Medjoul	5.74 ± 0.02	32.50 ± 1.11	4.39 ± 0.65	4.96 ± 1.04	6.63 ± 1.20	48.22 ± 2.04
Confitera	5.94 ± 0.04	32.36 ± 0.30	5.13 ± 0.35	5.85 ± 0.33	7.78 ± 0.36	48.72 ± 2.32
*p* value	0.003	0.732	0.009	0.028	0.014	0.640

**Table 3:** Proximate composition (mg/100 g) of Spanish-grown Medjoul and Confitera date fruits.

Date cv.	Moisture	Protein	Fat	TDF ^1^	Ash	Sugars
Medjoul	32.38 ± 0.52	1.98 ± 0.05	0.37 ± 0.05	20.05 ± 0.81	2.05 ± 0.07	43.17 ± 0.67
Confitera	25.65 ± 0.65	2.58 ± 0.18	0.18 ± 0.04	21.94 ± 0.94	2.06 ± 0.03	47.59 ± 0.76
*p* value	0.000	0.032	0.009	0.291	0.820	0.043

^1^ TDF: Total Dietary Fiber.

**Table 4:** Mineral content (mg/100 g dry weight) of Spanish-grown Medjoul and Confitera date fruits.

Component	Medjoul	Confitera	*p* Value
K	639.39 ± 47.67	837.33 ± 68.82	0.013
Mg	89.82 ± 5.37	114.65 ± 7.41	0.009
Ca	100.62 ± 5.50	98.65 ± 1.72	0.325
Na	30.68 ± 0.61	43.15 ± 0.85	0.031
Fe	1.20 ± 0.01	1.70 ± 0.30	0.024
Zn	0.83 ± 0.08	0.99 ± 0.09	0.019
Cu	0.65 ± 0.07	0.62 ± 0.03	0.287
Mn	0.39 ± 0.02	0.50 ± 0.01	0.037

**Table 5:** Polyphenol composition (μg/g) of Spanish-grown Medjool and Confitera date fruits.

Compound	Medjoul	Confitera	*p* Value
(−) Epicatechin	LOD	1241.90 ± 1.13	-
Luteolin-7-O-glucoside	26.61 ± 0.74	8.21 ± 0.47	0.012
(+) Catechin	LOD	1150.89 ± 1.86	-
Caffeic acid	3.37 ± 0.09	3.32 ± 0.06	0.231
Eriocitrin	8.76 ± 0.23	10.85 ± 0.12	0.027
Hesperidin	7.47 ± 0.32	16.76 ± 0.41	0.013
Rutin	11.00 ± 0.48	12.97 ± 0.63	0.042
Isoquercitrin	19.03 ± 0.74	10.43 ± 0.36	0.031

LOD: lower than limit of detection.

**Table 6:** Antioxidant properties of Spanish-grown Medjoul and Confitera date fruits.

Date cv.	FRAP (mg TE/g Pulp)	DPPH (μg TE/g Pulp)	FIC (μg EDTA/g Pulp)	ABTS (mg TE/g Pulp)
Medjoul	0.64 ± 0.05	26.46 ± 2.11	3.75 ± 0.39	1.14 ± 0.28
Confitera	0.78 ± 0.06	32.33 ± 3.13	4.63 ± 0.87	0.91 ± 0.14
*p* value	0.001	0.003	0.046	0.097

TE: Trolox Equivalents.

## Data Availability

Data are available upon request to the authors.

## References

[1] Fernández-López J., Viuda-Martos M., Sayas-Barberá E., Navarro-Rodríguez de Vera C., Pérez-Álvarez J.Á. (2022). Biological, nutritive, functional and healthy potential of date palm fruit (*Phoenix dactylifera* L.): Current research and future prospects. Agronomy.

[2] Echegaray N., Pateiro M., Gullón B., Amarowicz R., Misihairabgwi J., Lorenzo J.M. (2020). *Phoenix dactylifera* products in human health—A review. Trends Food Sci. Technol..

[3] Echegaray N., Gullón B., Pateiro M., Amarowicz R., Misihairabgwi J., Lorenzo J.M. (2021). Date fruit and its by-products as promising source of bioactive components: A review. Food Rev. Int..

[4] FAO (2022). FAO Statistical Database (FAOSTAT).

[5] Falade-Kolawole O., Abbo Emmanuel S. (2007). Air-drying and rehydration characteristics of date palm (*Phoenix dactylifera* L.) fruits. J. Food Eng..

[6] Özlem E., Yeliz İ. (2020). Modeling of drying processes of dates (*Phoenix*, *Arecaceae*) with oven or TGA and microbiological properties of fresh and dried dates. Int. J. Fruit Sci..

[7] Abu-Reidah I.M., Gil-Izquierdo A., Medina S., Ferreres F. (2017). Phenolic composition profiling of different edible parts and by-products of date palm (*Phoenix dactylifera* L.) by using HPLC-DAD-ESI/MSn. Food Res. Int..

[8] Salomón-Torres R., Ortiz-Uribe N., Valdez-Salas B., Rosas-González N., García-González C., Chávez D., Córdova-Guerrero I., Díaz-Rubio L., Haro-Vázquez M.d.P., Mijangos-Montiel J.L. (2019). Nutritional assessment, phytochemical composition and antioxidant analysis of the pulp and seed of medjool date grown in Mexico. Peer J..

[9] El-Far A.H., Oyinloye B.E., Sepehrimanesh M., Allah M.A.G., Abu-Reidah I., Shaheen H.M., Razeghian-Jahromi I., Noreldin A.E., Al Jaouni S.K., Mousa S.A. (2019). Date palm (*Phoenix dactylifera*): Novel findings and future directions for food and drug discover. Curr. Drug Discov. Technol..

[10] El Arem A., Saafi E.B., Lahouar L., Bakhrouf A., Hammami M., Achour L. (2017). Antibacterial activity and principal analysis of chemical composition and antioxidant activity of Tunisian date palm (*Phoenix dactylifera* L.) fruit during ripening. J. Bioresour. Valoriz..

[11] Taleb H., Maddocks S.E., Morris R.K., Kanekanian A.D. (2016). Chemical characterization and the anti-inflammatory, anti-angiogenic and antibacterial properties of date fruit (*Phoenix dactylifera* L.). J. Ethnopharmacol..

[12] Hussain M.I., Farooq M., Syed Q.A. (2020). Nutritional and biological characteristics of the date palm fruit (*Phoenix dactylifera* L.)—A review. Food Biosci..

[13] Eid N.M.S., Al-Awadi B., Vauzour D., Oruna-Concha M.J., Spencer J.P.E. (2013). Effect of cultivar type and ripening on the polyphenol content of date palm fruit. J. Agric. Food Chem..

[14] Al-Qarni S.S.M., Bazzi M.D. (2020). Date fruit ripening with degradation of chlorophylls, carotenes, and other pigments. Int. J. Fruit Sci..

[15] López-Marcos M.C., Bailina C., Viuda-Martos M., Pérez-Alvarez J.A., Fernández-López J. (2015). Properties of dietary fibers from agroindustrial coproducts as source for fiber-enriched foods. Food Bioprocess Technol..

[16] AOAC (2019). Official Methods of Analysis of AOAC International.

[17] Melgarejo-Sánchez P., Martínez J.J., Legua L., Martínez R., Hernández F., Melgarejo P. (2015). Quality, antioxidant activity and total phenols of six Spanish pomegranates clones. Sci. Hortic..

[18] Genskowsky E., Puente L.A., Pérez-Álvarez J.A., Fernández-López J., Muñoz L.A., Viuda-Martos M. (2016). Determination of polyphenolic profile, antioxidant activity and antibacterial properties of maqui [*Aristotelia chilensis* (Molina) Stuntz] a Chilean blackberry. J. Sci. Food Agric..

[19] Brand-Williams W., Cuvelier M.E., Berset C. (1995). Use of a free radical method to evaluate antioxidant activity. LWT Food Sci. Technol..

[20] Gullón B., Pintado M.E., Fernández-López J., Pérez-Álvarez J.A., Viuda-Martos M. (2015). In vitro gastrointestinal digestion of pomegranate peel (*Punica granatum*) flour obtained from co-products: Changes in the antioxidant potential and bioactive compounds stability. J. Funct. Foods.

[21] Oyaizu M. (1986). Studies on products of browning reaction: Antioxidative activity of products of browning reaction prepared from glucosamine. Jpn. J. Nutr..

[22] Mahdavi B., Yaacob W.A., Din L.B. (2017). Chemical composition, antioxidant, and antibacterial activity of essential oils from Etlingera sayapensis A.D. Poulsen & Ibrahim. Asian Pac. J. Trop. Med..

[23] Martín-Sánchez A.M., Vilella-Esplá J., Palou L.l., del Río M.A., Ben Abda J., Sayas-Barberá E., Pérez-Alvarez J.A. (2010). Criterios técnicos de dátiles comercializados en España. Aliment. Equipos Tecnol..

[24] Muralidhara B.M., Singh R.S., Bhargava R., Veena G.L., Kumar M.K. (2016). Morphological characterization of date fruits at different growth stages under hot arid conditions. Environ. Ecol..

[25] Al-Qurashi A.D. (2010). Physico-chemical changes during development and ripening of Helali date palm fruit. J. Food Agric. Environ..

[26] Awad M.A., Al-Qurashi A.D., Mohamed S.A. (2011). Biochemical changes in fruit of an early and a late palm cultivar during development and ripening. Int. J. Fruit Sci..

[27] Meligi M.A., Sourial G.F. Fruit Quality and General Evaluation of Some Iraqi Date Palm Cultivars Grown under Conditions of Barrage Region. Proceedings of the First Symposium on the Date Palm Conference.

[28] Al-Hoots S., Sidhu J.S., Qabazard H. (1997). Physicochemical characteristics of five date fruit cultivars grown in the United Arab Emirates. Plant Foods Hum. Nutr..

[29] Borchani C., Besbes S., Blecker C., Masmoudi M., Baati R., Attia H. (2010). Chemical properties of 11 date cultivars and their corresponding fiber extract. Afr. J. Biotechnol..

[30] Almeida J., Assis R., Molineri V.N., Sestari I., Lira B.S., Carrari F., Peres L.E., Rossi M. (2015). Fruits from ripening impaired, chlorophyll degraded and jasmonate insensitive tomato mutants have altered tocopherol content and composition. Phytochemistry.

[31] Ghnimi S., Umer S., Karim A., Kamal-Eldin A. (2017). Date fruit (*Phoenix dactylifera* L.): An underutilized food seeking industrial valorization. NFS J..

[32] Al-Okbi S.Y. (2022). Date palm as source of nutraceuticals for health promotion: A review. Curr. Nutr. Rep..

[33] Hazbavi I., Khoshtaghaza M.H., Mostaan A., Banakar A. (2015). Effect of postharvest hot-water and heat treatment on quality of date palm (cv. Stamaran). J. Saudi Soc. Agric. Sci..

[34] IRANOR (1981). Nomenclatura Cromática Española.

[35] Dhingra D., Michael M., Rajput H., Patil R.T. (2012). Dietary fibre in foods: A review. J. Food Sci. Technol..

[36] Sahni P., Shere D.M. (2017). Comparative evaluation of physico-chemical and functional properties of apple, carrot and beetroot pomace powders. Int. J. Food Ferment. Technol..

[37] Viuda-Martos M., López-Marcos M.C., Fernández-López J., Sendra E., López-Vargas J.H., Pérez-Alvarez J.A. (2010). Role of fiber in cardiovascular diseases: A review. Compr. Rev. Food Sci. Food Saf..

[38] Sharoba A.M., Farrag M.A., Abd El-Salam A.M. (2013). Utilization of some fruits and vegetables waste as a source of dietary fiber and its effect on the cake making and its quality attributes. J. Agroalim. Process. Technol..

[39] Sánchez-Zapata E., Fernández-López J., Peñaranda M., Fuentes-Zaragoza E., Sendra E., Sayas E., Pérez-Alvarez J.A. (2011). Technological properties of date paste obtained from date by-products and its effect on the quality of a cooked meat product. Food Res..

[40] Chia S.L., Chong G.H. (2015). Effect of drum drying on physico-chemical characteristics of dragon fruit peel (*Hylocereus polyrhizus*). Int. J. Food Eng..

[41] Jasim A., Almusallam A., Al-Hooti S.N. (2012). Isolation and characterization of insoluble date (*Phoenix dactylifera* L.) fibers. LWT Food Sci. Technol..

[42] Mrabet A., Hammadi H., Rodríguez-Gutierrez G., Jiménez-Araujo A., Sindic M. (2019). Date palm fruits as a potential source of functional dietary fiber: A review. Food Sci. Technol. Res..

[43] Majzoobi M., Karambakhsh G., Golmakani M.T., Mesbahi G.R., Farahnaky A. (2019). Chemical composition and functional properties of date press cake, an agro-industrial waste. J. Agric. Sci. Technol..

[44] Sánchez-Zapata E., Fuentes-Zaragoza E., Fernández-López J., Sendra E., Sayas E., Navarro C., Pérez-Alvarez J.A. (2009). Preparation of dietary fiber powder from tiger nut (*Cyperus esculentus*) milk (“Horchata”) byproducts and its physicochemical properties. J. Agric. Food Chem..

[45] Al-Farsi M.A., Lee C.Y. (2008). Nutritional and functional properties of dates: A review. Crit. Rev. Food Sci. Nutr..

[46] El Arem A., Saafi E.B., Flamini G., Issaoui M., Ferchichi A., Hammami M., Helall A.N., Achour L. (2012). Volatile and nonvolatile chemical composition of some date fruits (*Phoenix dactylifera* L.) harvested at different stages of maturity. Int. J. Food Sci. Technol..

[47] Vinita D.P. (2016). Nutritional composition of fruit of four date palm (*Phoenix dactylifera* L.) cultivars grown in Haryana, India. Asian J. Dairy Food Res..

[48] Martín-Sánchez A.M., Cherif S., Vilella-Esplá J., Ben-Abda J., Kuri V., Pérez-Álvarez J.Á., Sayas-Barberá E. (2014). Characterization of novel intermediate food products from Spanish date palm (*Phoenix dactylifera* L., cv. Confitera) co-products for industrial use. Food Chem..

[49] Tassoult M., Kati D.E., Fernández-Prior M.Á., Bermúdez-Oria A., Fernandez-Bolanos J., Rodríguez-Gutiérrez G. (2021). Antioxidant capacity and phenolic and sugar profiles of date fruits extracts from six different Algerian cultivars as influenced by ripening stages and extraction systems. Foods.

[50] Ashraf Z., Hamidi-Esfahani Z. (2011). Date and date processing: A review. Food Rev. Int..

[51] Rastegar S., Rahemir M., Bagihzadeg A., Gholami M. (2012). Enzyme activity and biochemical changes of three date palm cultivars with different softening pattern during ripening. Food Chem..

[52] Bouhlali E.d.T., Ramchoun M., Alem C., Ghafoor K., Ennassir J., Zegzouti Y.F. (2017). Functional composition and antioxidant activities of eight Moroccan date fruit varieties (*Phoenix dactylifera* L.). J. Saudi Soc. Agric. Sci..

[53] EFSA (European, Food Safety Authority) (2019). Dietary reference values for sodium. EFSA J..

[54] El Hadrami A., Daayf F., Elshibli S., Jain S.M., El Hadrami I., Jain S., Al-Khayri M.J.M., Johnson D.V. (2011). Somaclonal Variation in Date Palm. Date Palm Biotechnology.

[55] Nematallah K.H., Ayoub N.A., Abdelsattar E., Meselhy M.R., Elmazar M.M., El-Khatib A.H., Linscheid M.W., Hathout R.M., Godugu K., Adel A. (2018). Polyphenols LC-MS2 profile of Ajwa date fruit (*Phoenix dactylifera* L.) and their microemulsion: Potential impact on hepatic fibrosis. J. Funct. Foods.

[56] Hong Y.J., Tomas-Barberan F.A., Kader A.A., Mitchell A.E. (2006). The flavonoid glycosides and procyanidin composition of Deglet Noor dates (*Phoenix dactylifera*). J. Agric. Food Chem..

[57] Mrabet A., Jiménez-Araujo A., Fernández-Bolaños J., Rubio-Senent F., Lama-Muñoz A., Sindic M., Rodríguez-Gutiérrez G. (2016). Antioxidant phenolic extracts obtained from secondary Tunisian date varieties (*Phoenix dactylifera* L.) by hydrothermal treatments. Food Chem..

[58] Hachani S., Hamia C., Boukhalkhal S., Silva A.M., Djeridane A., Yousfi M. (2018). Morphological, physico-chemical characteristics and effects of extraction solvents on UHPLC-DAD-ESI-MSn profiling of phenolic contents and antioxidant activities of five date cultivars (*Phoenix dactylifera* L.) growing in Algeria. NFS J..

[59] Mansouri A., Embarek G., Kokkalou E., Kefalas P. (2005). Phenolic profile and antioxidant activity of the Algerian ripe date palm fruit (*Phoenix dactylifera*). Food Chem..

[60] Hamad I., AbdElgawad H., Al Jaouni S., Zinta G., Asard H., Hassan S., Selim S. (2015). Metabolic analysis of various date palm fruit (*Phoenix dactylifera* L.) cultivars from Saudi Arabia to assess their nutritional quality. Molecules.

[61] Abdul-Hamid N.A., Mustaffer N.H., Maulidiani M., Mediani A., Ismail I.S., Tham C.L., Shadid K., Abas F. (2020). Quality evaluation of the physical properties, phytochemicals, biological activities and proximate analysis of nine Saudi date palm fruit varieties. J. Saudi Soc. Agric. Sci..

[62] Allaith A.A.A. (2008). Antioxidant activity of Bahraini date palm (*Phoenix dactylifera* L.) fruit of various cultivars. Int. J. Food Sci. Technol..

[63] Al-Mssallem M.Q., Alqurashi R.M., Al-Khayri J.M., Murthy H., Bapat V. (2020). Bioactive Compounds of Date Palm (*Phoenix dactylifera* L.). Bioactive Compounds in Underutilized Fruits and Nuts.

